# Impact of Poly (Styrene–Acrylic Acid) Latex Nanoparticles on Colorectal and Cervical Cancer Cells

**DOI:** 10.3390/polym13132025

**Published:** 2021-06-22

**Authors:** Munther Alomari, Arwa Almahasheer, Balasamy Rabindran Jermy, Amal A. Al-Dossary, Hiba Bahmdan, Vijaya Ravinayagam, Deena Ababneh, Mohamad Tarhini, Abdelhamid Elaissari

**Affiliations:** 1 Department of Stem Cell Biology, Institute for Research and Medical Consultations (IRMC), Imam Abdulrahman Bin Faisal University, P.O. Box 1982, Dammam 34212, Saudi Arabia; AA-HHred@hotmail.com (A.A.); hbahmdan@iau.edu.sa (H.B.); 2 College of Science, Imam Abdulrahman Bin Faisal University, P.O. Box 1982, Dammam 34212, Saudi Arabia; 3 Department of Nano-Medicine Research, Institute for Research and Medical Consultations, Imam Abdulrahman Bin Faisal University, P.O. Box 1982, Dammam 34212, Saudi Arabia; rjermy@iau.edu.sa; 4 Department of Basic Sciences, Deanship of Preparatory Year and Supporting Studies, Imam Abdulrahman Bin Faisal University, P.O. Box 1982, Dammam 34212, Saudi Arabia; amaldossary@iau.edu.sa; 5 Deanship of Scientific Research, Department of Nano-Medicine Research, Imam Abdulrahman Bin Faisal University, Dammam 31441, Saudi Arabia; vrnayagam@iau.edu.sa; 6 Department of Basic Sciences and Humanities, College of Engineering, Imam Abdulrahman Bin Faisal University, P.O. Box 1982, Dammam 31441, Saudi Arabia; dababneh@iau.edu.sa; 7 CNRS, Univ Lyon, University Claude Bernard Lyon-1, ISA-UMR 5280, F-69622 Lyon, France; mohamad.tarhini@etu.univ-lyon1.fr (M.T.); abdelhamid.elaissari@univ-lyon1.fr (A.E.)

**Keywords:** poly (styrene–acrylic acid) latex nanoparticles, HCT-116, HELA, cancer, delivery

## Abstract

Polymer nanoparticles are a promising approach for cancer treatment and detection, due to their biocompatibility, biodegradability, targeting capabilities, capacity for drug loading and long blood circulation time. This study aims to evaluate the impact of poly (styrene–acrylic acid) latex particles on colorectal and cervical cancer cells for anti-tumor efficiency. Latex particles were synthesized by a surfactant-free radical emulsion polymerization process and the obtained polymer particles were characterized in terms of size, size distribution, morphology using scanning electron microscopy (SEM) and transmission electron microscopy (TEM), and electrokinetic property (i.e., zeta potential). Human colorectal and cervical cancer, and normal cell lines, were then treated with different concentrations of poly (styrene–acrylic acid) latex particles. The cell morphology changes were pointed out using an optical microscope and the nanoparticles’ (NPs) cell cytotoxicity was evaluated using MTT assay. The obtained results showed that poly (styrene–acrylic acid) latex particles are effective against colorectal and cervical cancer cells if treated with an appropriate particle concentration for 48 h. In addition, it showed that normal cells are the least affected by this treatment. This indicates that these NPs are safe as a drug delivery carrier when used at a low concentration.

## 1. Introduction

The main characteristic of malignant tumors is that the cells grow continuously and uncontrollably. A variety of treatment options, including radiation therapy, chemotherapy, targeted therapy and surgery, are used to prolong a patient’s survival with a better quality of life. Cancer remains the leading cause of death worldwide [1,2]. Colorectal cancer and cervical cancer are the most diagnosed cancers [3,4]. Colorectal cancer is considered the second most deadly cancer worldwide [5,6]. Despite notable progress in treatment, approximately 86% of colorectal cancer patients with advanced stage cancer die within five years of the diagnosis [7]. On the other hand, cervical cancer is the third most common cancer that affects women worldwide after breast and colorectal cancers, and a major cause of death from gynecological cancer [8,9]. To date, multidrug resistance (MDR) is one of the top challenges in clinical anti-tumor chemotherapy [10,11,12,13]. Furthermore, the delivery of chemotherapeutic drugs to target-specific sites is a major challenge in the available treatment of cancer, as only a small number of chemotherapy drugs may reach the target site [14,15,16].

Thus, there is an urgent need to develop and design novel therapeutic strategies to overcome these challenges. A variety of nano-drug delivery systems have been established for the systemic delivery of insoluble drugs, including microemulsions [17], micelles [18], lipid-based NPs [19], polymeric NPs [20], and liposomes [21]. Among these, polymeric NPs are ideal drug carriers for anti-tumor medicines [22,23,24]. The main advantage of using polymeric NPs is their good biocompatibility and sustained drug-release capabilities at the target site [25].

Polymeric NPs are a strong platform for cancer therapy where they can encapsulate both hydrophilic and hydrophobic drugs, and have drawn great attention due to their possible application as drug delivery vehicles [26], such as poly (styrene–acrylic acid) latex. Styrene is mostly used in copolymerization because of its chemical characteristic, hydrophobic property, translucence and low cost. In addition, acrylic acid possesses high reactive double bonds that add crucial properties, such as top stability and polarity in an aqueous manner for copolymers because the copolymer surface has carboxylic groups and hydrophilicity [27].

The emulsion polymerization process is a powerful tool for the production of nanosized polymeric NPs [28]. This process involves emulsification of hydrophobic monomers by an oil-in-water emulsifier, then reaction initiation with a water-soluble initiator (e.g., potassium persulfate (K_2_ S_2_ O_8_)) [29]. Photochromic latex containing 1′-(2-acryloxyethyl)-3′3′-dimethyl-6-nitrospiro-(2-H-1-benzopyran-2,2′-indoline (spiropyran ethyl acrylates) showed robust photoactivity under UV radiation [30]. Polyspiropyran methacrylate latex nanoparticles that have been reported using reversible addition–fragmentation chain transfer mediated emulsion polymerization exhibited a reversible stimuli property. Spiropyran with photochromic properties tends to interact with polymer and show photo/pH responsivity [31]. Copolymer nanolatex based on poly (methyl methacrylate-co-N-vinyl caprolactam) synthesized by a microemulsion technique exhibited a thermosensitive property [32]. SPIONs/Poly (N-isopropylacrylamide) with amine functionalization in the form of microgel was reported to encapsulate polyphenol (curcumin) efficiently and trigger release under a magnetic field [33]. Poly (N-isopropylacrylamide)-based polymers are studied for their thermosensitive characteristics, exhibiting a lower consolute temperature at about 32 °C. Polymer nanogel with a triple environmental (temperature/pH/redox) responsive P (N-isopropylacrylamide–N,N′-dimethylaminoethyl methacrylate and N,N′-bis(acryloyl)cystamine (PND-BAC) nanoparticles has been reported as an effective nanocarrier for DOX release [34].

Surfactant-free emulsion polymerization has been reported to be an attractive technique to prepare colloidal microspheres. Styrene with various functional modalities has been effectively used to prepare monodisperse microspheres [35]. Magnetic submicronic latexes with superparamagnetic properties have been reported using styrene and divinylbenzene as cross-linker [36] for shell preparation. Acrylic acid possesses high polymerization reactivity in water and leads when copolymerized with styrene to submicron monodisperse particles. The molar ratio of acrylic acid over styrene leads to controlling the particle size. The particles obtained from such a process, using styrene/acrylic acid, exhibit good colloidal stability against salinity and hydrophilic particle surface, induced by the hydrophilic character of the carboxylic group of acrylic acid [27,37]. This study shows that the polydispersity index is generally close to one, indicating uniform submicron particle size distributions. In addition, irrespective of the polydispersity index, the latex particle size of the styrene/acrylic acid system was found to be inversely proportional to the acrylic acid concentration in the polymerization medium [37,38].

This work aims to investigate the potential of using poly (styrene–acrylic acid) latex particles P(St/AA) on colorectal and cervical cancer cells by studying various parameters. These surfactant-free P(St/AA) latex particles were specially synthesized by batch emulsifier-free emulsion polymerization in the presence of an anionic radical initiator (potassium persulfate).

## 2. Materials and Method

Styrene monomer (St, Mw 104.15 g/mol), anhydrous acrylic acid (AA, Mw 72.06 g/mol) and potassium persulfate (KPS, Mw 270.322 g/mol) were purchased from Fluka (Fisher Scientific, Illkirch, France). Monomers were purified by distillation under reduced pressure before being stored at −20 °C. KPS, sodium chloride (NaCl, Merck, Analytical, Merck KGaA, Darmstadt, Germany), sodium hydroxide (NaOH, VWR, BDH, Prolabo) and hydrochloric acid (HCl, VWR, BDH, Prolabo) were used as received. Deionized water (milli-Q) was used throughout the work.

### 2.1. Preparation of Polystyrene Particles

Surfactant-free emulsion copolymerization of styrene and acrylic acid, in the presence of potassium persulfate (KPS) as an initiator, was performed in a glass reaction vessel equipped with a mechanical stirrer, condenser, and nitrogen inlet and outlet. Deionized water (190 g) was charged into a 200 mL glass reaction vessel, while the remaining water was used to dissolve the KPS initiator. After purging with nitrogen for about 1 h while stirring at 350 rpm, the distilled St (20 g) and AA (2 g) monomers were added into the reactor. The polymerization temperature was controlled at 70 °C by using an external batch water circulation. The reaction was started by adding KPS (0.1 g) dissolved in 10 g of deionized water. Polymerization was carried out for 12 h and the polymerization conversion was determined gravimetrically.

### 2.2. Particle Size, Distribution, and Morphology

Particle size was measured both by Quasi Elastic Light Scattering (QELS) and by Transmission Electron Microscopy (TEM). The QELS was investigated using NanoZS from Malvern Instruments (Malvern, UK). The measurements were performed at least four times and the reported values are the average values. TEM analysis was investigated using a Hitachi S 800 (Hitachi High-tech, Europe GmbH, Paris, France). This method gives information on the particle size and distribution of latexes under a dried state. Samples for TEM were prepared by placing a drop of the dispersion directly onto a grid and drying the latex at room temperature. SEM was performed using a FEI Quanta 250 FEG microscope (FEI Europe, Amsterdam, The Netherlands) at the “Centre Technologique des Microstructures” (CTµ) at the University of Lyon (Villeurbanne, France). A drop of the diluted aqueous suspension of nanoparticles was deposited on a flat steel holder and dried at room temperature. The sample was finally coated under vacuum by cathodic sputtering with copper. The samples were observed by SEM under an accelerating voltage of 15 kV.

### 2.3. Electrokinetic Study

The electrophoretic mobility of latexes was measured using the NanoZS from Malvern Instruments (Malvern, UK). The experiments were carried out using highly diluted latex particles in a 10^−3^ M NaCl concentration at a given pH. The electrophoretic mobilities were determined as a function of pH at 20 °C. Each value was obtained by taking the average of at least three measurements.

### 2.4. Cell Culture and Nanoparticle Treatments

Human colon cancer cells (HCT-116, ATCC^®^ CCL-247™), human cervical cancer cells (HELA, ATCC^®^ CCL-2™) and normal human foreskin fibroblast cells (HFF-1, ATCC^®^ SCRC-1041™) were used in this study. The cells were sub-cultured in DMEM media, supplemented with 1% L-glutamine, 10% fetal bovine serum (FBS), 1% penicillin/streptomycin (HyClone, GE Healthcare, Chicago, IL, USA), and maintained in a humidified incubator (Thermo Scientific, Waltham, MA, USA) with 5% CO_2_ at 37 °C. When the cells achieved 80% confluence, they were sub-cultured in 96-well plates (1.5 × 10^4^/well). The cells were then treated with different concentrations of poly (styrene–acrylic acid) latex nanoparticles (0, 25, 50, 100, 200, 400 µg/mL) for 24 h and 48 h of treatment with 5% CO_2_ at 37 °C.

### 2.5. MTT Assay

MTT (3-(4,5-Dimethylthiazol-2-yl)-2,5-Diphenyltetrazolium Bromide) assay is a dye reduction test, used as an indicative of cell viability. Cell viability was measured by the ability of the living cells to convert yellow-colored MTT (tetrazolium dye) into a purple-colored formazan dye that can be detected spectrophotometrically. The exponentially growing HCT-116 and HELA cells were seeded in 96-well plates at an initial density of 1.5 × 10^4^/well, treated with different concentrations (0, 25, 50, 100, 200, 400 µg/mL) of newly synthesized poly (styrene-acrylic acid) latex particles, and maintained for 24 h at 37 °C in a 5% CO_2_ incubator (Thermo Scientific, Waltham, MA, USA). The media were then carefully removed from the wells, and the cells were incubated in 20 µL of MTT (Sigma-Aldrich, St Louis, MO, USA) at a concentration of 10 mg/mL in phosphate buffer saline (PBS) for 3 h at 37 °C. Formazan dye that crystalized in live cells was solubilized by 100 µL of isopropanol and 0.04% HCl for 1 h at 37 °C and measured in a microtiter plate reader (Tecan Infinite 200 PRO, Geneva, Switzerland) at 570 nm. Cell proliferation was expressed as a percentage of cell viability of those treated relative to the untreated control.

### 2.6. Morphological Characterization of Nanoparticle-treated HCT-116 and HELA Cells

The cytomorphological changes of treated HCT-116 and HELA cells and the control were studied under an optical microscope (TS100F Eclipse, Nikon, Tokyo, Japan). The HCT-116 cells were treated with different concentrations (0, 25, 50, 100, 200, 400 µg/mL) of newly synthesized poly (styrene–acrylic acid) latex particles. The treated cells were incubated for 24 h and 48 h at 37 °C in a 5% CO_2_ incubator (Thermo Scientific, Waltham, MA, USA). After treatment, the morphological changes were observed under an optical microscope.

### 2.7. Statistical Analysis

The obtained results were expressed as mean ± standard deviation (SD), and MTT data were analyzed with a *t*-test and one-way analysis of variance (ANOVA). The difference was considered statistically significant at *p* < 0.05. All experiments (*n* = 3) were carried out in triplicate.

## 3. Results and Discussion

### 3.1. Characterization of Synthesized Poly (Styrene–Acrylic Acid) Latex Particles

The importance of nanoparticle size in cancer treatment is heavily studied. The size of the administered particles can affect the biodistribution, in that particles with certain size intervals are more likely to accumulate in specific organs. Moreover, particle size has a direct effect on their clearance. Particles with a diameter larger than 200 nm are more likely to be cleared by the mononuclear phagocytic system [39].

**Figure 1:**
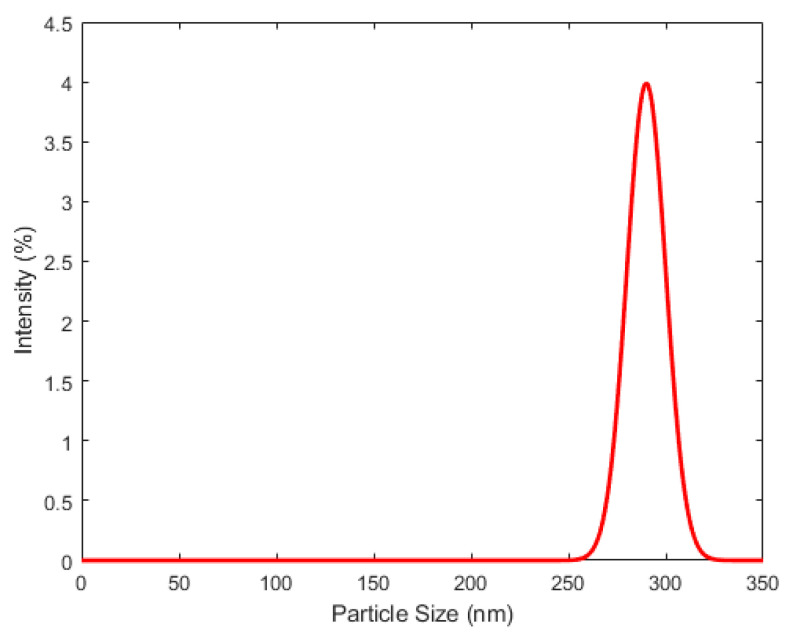
Hydrodynamic size of P(St/AA) latex particles measured in 1 mM NaCl and at 20 °C.

Using QELS, the hydrodynamic size of P(St/AA) particles was measured in 1 mM Sodium Chloride at 20 °C. It was found that the average hydrodynamic particle size is around 280 nm (+/−10 nm), and the obtained size distribution is reported in Figure 1. The observed narrow size distributed, reported in Figure 1, shows good monodispersity of the latex particles.

Additionally, the surface charge of the particles was proven to be around −50 mV. This high negativity will lead to a long-term stability of the particles in the dispersed medium due to electrostatic repulsion.

**Figure 2:**
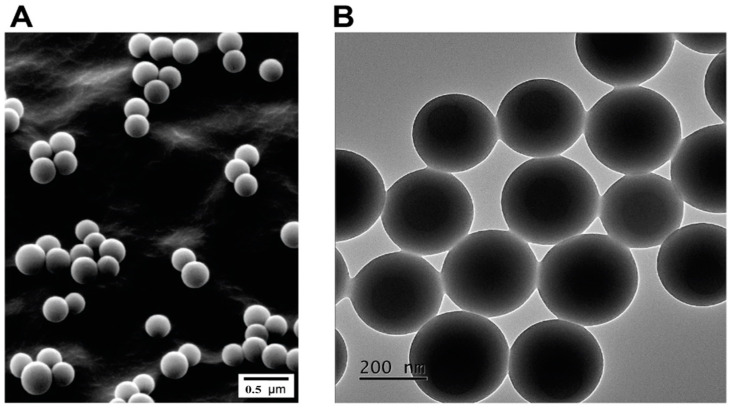
(**A**) Scanning electron microscopy image of P(St/AA) particles. (**B**) Transmission electron microscopy images of P(St/AA) particles.

The morphology of the particles was examined using both scanning and transmission electron microscopy (SEM) and transmission electron microscopy (TEM), as shown in Figure 2A,B, respectively. It was found that the particles are spherical with a smooth surface. As can be seen from Figure 2B, the average particle size from TEM is around 275 nm; slightly lower than the hydrodynamic size as generally reported. This difference is attributed to the difference between the analysis in dispersed media based on Brownian motion and the measurement of the dried sample by TEM.

**Figure 3:**
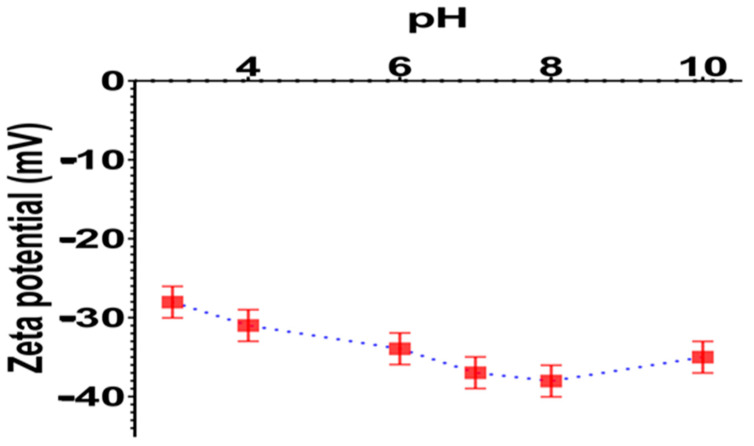
The zeta potential of P(St/AA) particles as a function of pH at 20 °C.

The zeta potential of P(St/AA) latex particles was determined by measuring the electrophoretic mobility as a function of pH at 20 °C. The measured electrophoretic mobilities are transformed to Smoluchowski’s zeta potential. Figure 3 represents the variation of the zeta potential as a function of the pH in 1 mM NaCl solution. As expected, the deduced zeta potential was found to be negative in the pH range between pH 3 and 10, revealing that the particles are negatively charged. This surface charge is originated from initiator fragments (sulfate compounds) and carboxylic acid from the acrylic acid monomer. It is interesting to notice that the sulfate group is a strong acid and consequently, its dissociation is pH-independent, and particles bearing only such groups exhibit negative and constant zeta pH 3. The observed slight increase in zeta potential as a function of pH can be attributed to carboxylic groups from the acrylic acid monomer.

The absolute zeta potential values above 20 mV revealed a high surface charge and consequently guaranteed good colloidal stability.

### 3.2. In Vitro MTT Assay of Synthesized Poly (Styrene–Acrylic Acid) Latex Particles

**Figure 4:**
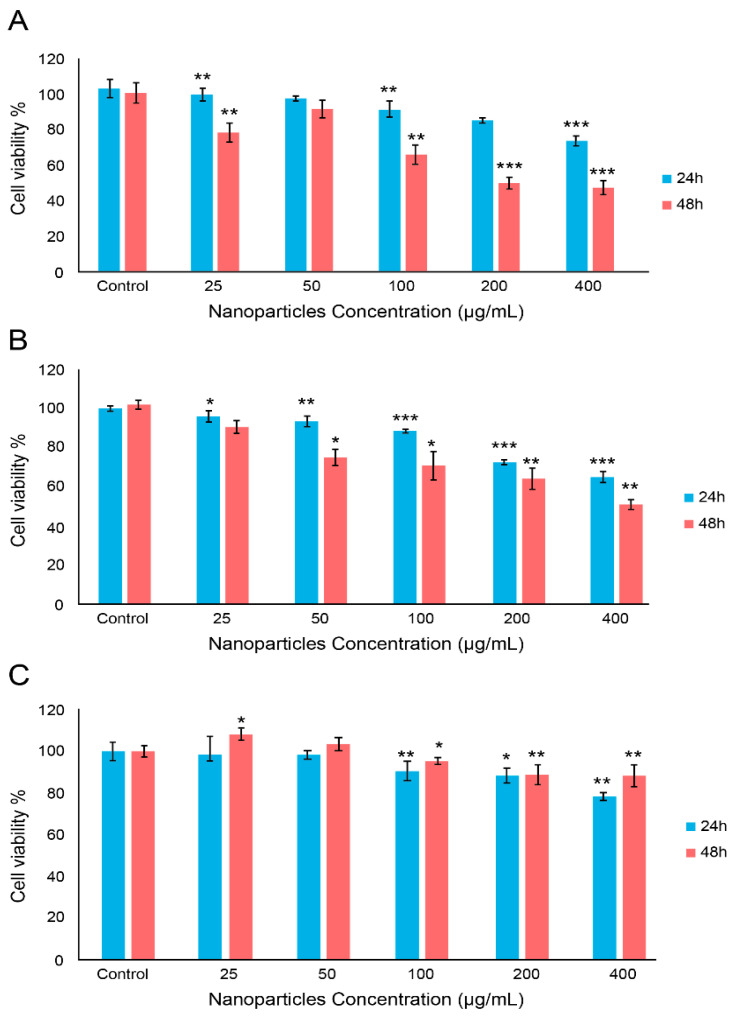
The percentage of viable cells of (**A**) HCT-166; (**B**) HELA; and (**C**) HFF-1 cells treated with synthesized poly (styrene–acrylic acid) latex particles at different concentrations. Data are the means ± standard deviation (SD). The *t*-test was performed on three independent sets of experiments conducted in triplicate. * *p* values < 0.05. ** *p* values < 0.01. *** *p* values < 0.001.

MTT assays were performed on human colon cancer cells (HCT-116, ATCC^®^ CCL-247™), cervical cancer cells (HELA, ATCC^®^ CCL-2™) and normal foreskin fibroblast cells (HFF-1, ATCC^®^ SCRC-1041™). The results showed that, for HCT cells, the 24 h treatment shows a decrease in cell viability starting from a 25 µg/mL P(St/AA) particle concentration. The decrease continues with the increasing number of nanoparticles to reach viability of around 75% at 400 µg/mL. The 48 h treatment follows the same pattern, with higher efficacy. At 400 µg/mL the cell viability reaches 52% at 400 µg/mL (Figure 4A). Similar behavior is observed with HELA cells (Figure 4B).

In the case of normal foreskin fibroblast cells HFF-1, for both 24 and 48 h treatment, no significant effect was observed on cell viability with nanoparticle concentrations of 25 and 50 µg/mL. However, a slight decrease in cell viability can be observed for a nanoparticle concentration higher than 100 µg/mL. At 400 µg/mL the cell viability reaches 90% at 48 h and around 85% at 24 h treatment (Figure 4C). These results show the efficacy of the P(St/AA) particles against colorectal and cervical cancer cells, and their safety against the normal cell.

**Figure 5:**
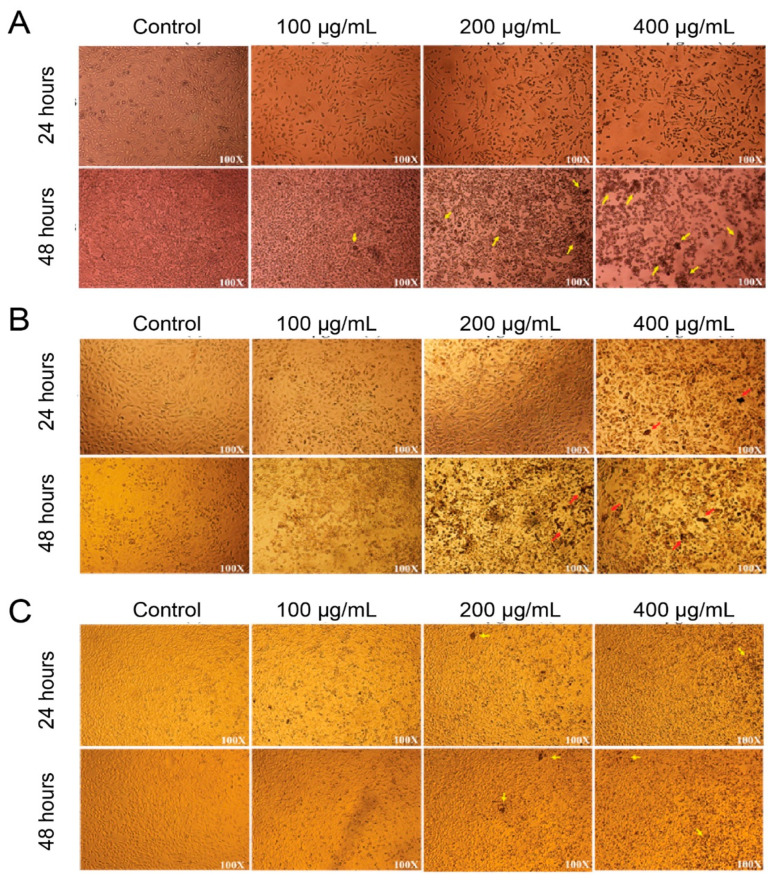
Microscopic images (×100 magnification) of nanoparticle-treated (**A**) HCT-116 colon cancer cells; (**B**) HELA; and (**C**) HFF-1 cells, for 24 h and 48 h with different P(St/AA) particle concentrations. The small yellow or red arrows indicate the brown debris of the dead cells.

For better confirmation, images of the three cell cultures were taken by optical microscopy after treatment with nanoparticles (Figure 5). The increase in the amount of brown debris with the increase in nanoparticle concentration can be noted. The debris was mostly observed in the case of HCT-116 colon cancer cells (Figure 5A). It was also observed in a significant amount with HELA cells (Figure 5B). This debris is present in a minimal amount in the case of normal cells (Figure 5C). These images confirm the results of the MTT assay.

The selectivity of P(St/AA) particles towards cancer cells can be attributed first to the characteristics of cancer cells. Tumor cells often possess a unique permeability that, in addition to the optimal particle size (20–200 nm), can increase the anti-cancer activity of the particles. In addition, cancer cells usually have a higher receptor expression compared to normal cells [40]. Therefore, the improved efficacy of P(St/AA) particles against cancer cell lines can possibly be attributed to: (i) as the fetal bovine serum is present in the cell culture medium (DMEM), the possible adsorption of FBS on P(St/AA) [41] may lead to the screening of particle surface charge density and a biocompatible surface and (ii) the presence of carboxylic groups induced by acrylic acid monomer can also induce cell–particle interactions via hydrogen bonding, and consequently improve the cellular uptake. In general, P(St/AA) particles were proven to be a promising candidate against cancer cells in terms of toxicity and specificity. Using these particles as a vehicle for anti-cancer drugs can increase the efficacy of the latter through better targeting and synergistic effect.

## 4. Conclusions

The prepared surfactant-free carboxylic-containing particles were prepared and well characterized in terms of physical chemistry. These particles are negatively charged and of high colloidal stability, irrespective of the incubation pH. The effect of particle amount on cell viability via MTT assays was assessed on human colon cancer cells (HCT-116, ATCC^®^ CCL-247™), cervical cancer cells (HELA, ATCC^®^ CCL-2™) and normal foreskin fibroblast cells (HFF-1, ATCC^®^ SCRC-1041™). The obtained results are encouraging, since they point out that the prepared surfactant-free particles were investigated, and the obtained results show that P(St/AA) nanoparticles have a marked effect on human colon cancer cells (HCT-116, ATCC^®^ CCL-247™) and cervical cancer cells (HELA, ATCC^®^ CCL-2™). The results show that poly (styrene–acrylic acid) latex nanoparticles are effective against colorectal and cervical cancer cells if treated with appropriate particle concentrations and for 48 h. Consequently, surfactant-free poly (styrene–acrylic acid) latex indicates that these submicron polymer particles are (i) safe as a carrier in drug delivery when used at low concentrations and (ii) are promising for treating or killing cancerous cells.

## Data Availability

Data is contained within the article.
